# Diagnostic accuracy of susceptibility-weighted magnetic resonance imaging for the evaluation of pineal gland calcification

**DOI:** 10.1371/journal.pone.0172764

**Published:** 2017-03-09

**Authors:** Lisa C. Adams, Sarah M. Böker, Yvonne Y. Bender, Gerd Diederichs, Eva M. Fallenberg, Moritz Wagner, Bernd Hamm, Marcus R. Makowski

**Affiliations:** Department of Radiology, Charité, Berlin, Germany; Henry Ford Health System, UNITED STATES

## Abstract

**Objectives:**

To determine the diagnostic performance of susceptibility-weighted magnetic resonance imaging (SWMR) for the detection of pineal gland calcifications (PGC) compared to conventional magnetic resonance imaging (MRI) sequences, using computed tomography (CT) as a reference standard.

**Methods:**

384 patients who received a 1.5 Tesla MRI scan including SWMR sequences and a CT scan of the brain between January 2014 and October 2016 were retrospectively evaluated. 346 patients were included in the analysis, of which 214 showed PGC on CT scans. To assess correlation between imaging modalities, the maximum calcification diameter was used. Sensitivity and specificity and intra- and interobserver reliability were calculated for SWMR and conventional MRI sequences.

**Results:**

SWMR reached a sensitivity of 95% (95% CI: 91%-97%) and a specificity of 96% (95% CI: 91%-99%) for the detection of PGC, whereas conventional MRI achieved a sensitivity of 43% (95% CI: 36%-50%) and a specificity of 96% (95% CI: 91%-99%). Detection rates for calcifications in SWMR and conventional MRI differed significantly (95% versus 43%, p<0.001). Diameter measurements between SWMR and CT showed a close correlation (R^2^ = 0.85, p<0.001) with a slight but not significant overestimation of size (SWMR: 6.5 mm ± 2.5; CT: 5.9 mm ± 2.4, p = 0.02). Interobserver-agreement for diameter measurements was excellent on SWMR (ICC = 0.984, p < 0.0001).

**Conclusions:**

Combining SWMR magnitude and phase information enables the accurate detection of PGC and offers a better diagnostic performance than conventional MRI with CT as a reference standard.

## Introduction

To the present day, the functions of the pineal gland are not fully understood. Unlike most parts of the brain, it lies outside the blood-brain barrier and is not separated from the bloodstream. Current knowledge indicates that by secretion of melatonin, the pineal gland plays an important role in the regulation of the sleep-wake cycle and of reproductive function (e.g. onset of puberty) [1], with melatonin also acting as a neuroprotector or antioxidant [2, 3]. Previous studies have suggested a decline of melatonin secretion with age and an association between melatonin decrease and neurodegenerative diseases such as Alzheimer’s or Parkinson’s disease [4–7]. The amount of uncalcified pineal tissue was shown to predict total melatonin excretion with lack of melatonin being hypothesized to result from pineal gland calcification (PGC) [8, 9]. As a consequence, detection and measurement of PGC might be of clinical interest by identifying patients with possible melatonin deficits and a risk for the development of neurodegenerative diseases [8, 9].

Another aspect is, that a wide range of lesions from different entities arises in the pineal region, which can be classified into tumors of germ cell origin, tumors of pineal cell origin and other tumors, and makes up for approximately 1% of intracranial tumors [10, 11]. Calcification of pineal region tumors is very common and tumor-specific patterns of calcification have been reported [11–13]. In germinomas, pineal calcifications tend to be engulfed by the tumor, whereas in pineoblastomas calcification is often not central, but “exploded” to the periphery [12]. As a consequence, the assessment of calcification in the pineal region might also have a clinical benefit by narrowing differential diagnosis of pineal region tumors.

PGC, also referred to as “brain sand”, involves the development of hydroxyapatite deposits and is very common with a reported prevalence of approximately 68–75% in adults [14–16]. It is frequently detected on computed tomography (CT) scans. As CT causes substantial radiation exposure, it would be of advantage to identify PGC with MRI instead. Apart from the absence of ionizing radiation, MRI provides superior soft-tissue contrast and is the modality of choice to evaluate the pineal region, as it enables an accurate delineation of pineal tumors before surgery. However, in the case of calcifications of the pineal gland or tumor calcifications in the pineal region, conventional MRI sequences do not allow for a reliable identification and have a poor sensitivity, as calcifications appear hypointense on T1, T2 and T2*weighted sequences and consequently cannot be reliably differentiated from e.g. soft tissue artifacts or microbleeds.

Recent advances in MRI imaging have led to the development of novel gradient echo (GRE) imaging techniques such as SWMR, which is based on magnetic susceptibility and sensitive to materials distorting the local magnetic field. SWMR allows for a reliable differentiation of calcifications from tissue artifacts, hemorrhage and other causes of susceptibility differences by using T2* weighted magnitude and GRE filtered-phase information to generate a unique contrast [17–21]. So far, SWMR, has mainly been used in neurovascular imaging, e.g. to detect and differentiate intracranial haemorrhage from calcifications, to visualize intracranial vessels, to identify intracranial thromboembolism or to evaluate cerebral metastases [19–24]. In recent studies the use of SWMR was also extended to extra-cranial imaging such as detection of calcific tendonitis [25] or the assessment of prostatic calcifications [26, 27]. With regard to intracranial calcifications there have been publications on vascular and tumor-associated calcifications, but not on calcifications of the pineal gland.

The purpose of the present study was to assess the diagnostic performance of SWMR for the evaluation of PGC, comparing it to conventional MRI sequences with CT as the reference standard.

## Materials and methods

### Study population

The local ethics committee approved this retrospective study, including a waiver of informed consent (approval number: EA1/385/16). Prior to analysis, all data were anonymized and de-identified. In total, 384 patients, who had received both a 1.5 Tesla MRI with SWMR sequences and a CT brain scan at the Department of Radiology at Charité University Hospital between January 2014 and October 2016, were investigated. The following exclusion criteria we defined: Age under 18 years (n = 22) and an interval of more than three months between CT and MRI examination (n = 16). CT imaging was performed with 64/128-slice scanners (Toshiba Medical Systems, Otawara, Japan) and MRI was conducted on 1.5 Tesla scanners (Aera/Avanto, Siemens Medical Solutions, Erlangen, Germany). In total, 346 patients could be included, of which 61.8% showed a calcification of the pineal gland on unenhanced CT brain scans. The mean age of the study population was 58.7 ± 17.4 (mean age ± standard deviation) with an age range of 18–95 (173 men with a mean age of 57.8 years ± 16.9, age range 21–88 years and 173 women with a mean age of 59.6 years ± 17.8, age range 18–95 years; difference in mean age values between men and women, p = 0.55). 132 patients without PGC were used for comparison and to evaluate sensitivity and specificity. In this group, the mean age was 55.7 ± 19.3 with an age range of 18–94 (59 men with a mean age of 56.3 years ± 17.6, age range 21–88 years and 73 women with a mean age of 55.3 years ± 20.7, age range 18–94 years; difference in mean age values between men and women, p = 0.77). Patients with pineal gland calcifications were significantly older than those without (mean age of 60.5 years versus 55.7 years, p = 0.017).

### Imaging protocol

CT, which was used as the standard of reference in the present analysis, was performed based on the following parameters (Toshiba Medical Systems): FOV of 220 x 220 mm, 1.0-mm slice thickness and a1.0 mm increment, 120 kV and 280 mAs. The AIDR 3D reconstruction algorithm was applied for iterative noise reduction. FC26 (Toshiba Medical Systems) was used as a standard reconstruction kernel for the head. A bone window was used for the analysis. MRI was performed with a 1.5 Tesla unit (Aera/Avanto, Siemens Medical Solutions, Erlangen, Germany) provided with a standard brain coil. According to a MRI protocol routinely used for clinical examinations, the following conventional sequences were acquired: axial T1-weighted turbo-spin-echo (TSE) images (field of view (FOV) of 230 × 230 mm, matrix of 256 x 256, repetition time of 550 ms, echo time of 8.4 ms, 23 slices, 5-mm slice thickness, 2 averages), axial T2-weighted TSE images (FOV of 230 × 230 mm, matrix of 256 x 256, repetition time of 3180 ms, echo time of 86 ms, 23 slices, 5-mm slice thickness, 2 averages), axial T2-weighted fluid-attenuated inversion recovery (FLAIR) images (FOV of 230 × 230 mm, matrix of 256 x 256, repetition time of 9000 ms, echo time of 89 ms, inversion time 2500 ms, 23 slices, 5-mm slice thickness, 1 average) and a sagittal magnetization-prepared rapid gradient-echo (MP-RAGE) sequence (FOV of 260 × 260 mm, matrix of 256 x 256, repetition time of 1940 ms, echo time of 2.91 ms, 192 slices, 1-mm slice thickness, 1 average).

For SWMR, a 3D fast low-angle gradient-echo sequence was performed. The imaging parameters of SWMR for brain imaging were as follows: FOV of 230 × 230 mm, matrix of 320 x 320, repetition time of 49 ms, echo time of 40 ms, 104 slices, 1.6-mm slice thickness, 1 average. SWMR images were created by a combination of magnitude and phase information [17, 18, 28]. While the magnitude image is calculated from a velocity-compensated three-dimensional GRE sequence to support detection of lesions with shortening of $T_{2}^{*}$ relaxation times [17, 18], phase images are computed in order to enable a differentiation between calcifications and other lesions such as tissue artefacts or microbleeds. Even though typical susceptibility weighted imaging (SWI) furthermore implies the generation of a phase mask, we found that for the visualization of larger calcifications and susceptibility changes, such as in the pineal gland, which is moreover surrounded by CSF, an approach using a combination of separate inverted magnitude and filtered phase images (SWMR) was better suited.

### Imaging analysis

SWMR, MRI and CT images were analyzed using PACS workstations (Centricity Radiology RA1000, GE Healthcare, United Kingdom). All CT, MRI and SWMR scans were assessed independently and in a randomized order by two radiologists with 1 (L.A.) and 4 (S.B) years of diagnostic experience. To determine calcifications on unenhanced CT scans, they were assessed based on their specific shape und density, whereby a threshold of 130 Hounsfield units (HU) was set [29]. Concerning conventional MRI, calcifications were more difficult to assess, as they appeared as focal hypointense areas in T1 and T2 weighted sequences. On SWMR sequences, PGCs were identified if they were located in the pineal region within the quadrigeminal cistern, showed a hyperintense signal intensity on the inverted magnitude image and a hyperintense signal on the filtered phase image [17, 18]. However, filtered phase images are more challenging to evaluate with regard to larger amounts of calcifications. Larger calcifications do not appear homogeneously hyperintense, but can show a hypointense rim or (central) areas that appear dark as a consequence of aliasing effects when the field is large enough so that the phase exceeds π radians [17, 30]. Calcification diameters were measured with electronic calipers on CT scans, MRI T2 weighted sequences and SWMR magnitude by two radiologists in order to assess interobserver and intermodality correlations. There was a washout period of two weeks between CT, MRI and SWMR assessment.

### Statistical analysis

Statistical analysis was performed using “R” Statistical Software (Version 3.2.2, R Development Core Team, 2015). Variables were expressed as means ± standard deviations. 95% confidence intervals were calculated. To determine the p-values for sex-related differences, two-tailored t-tests were applied. Sensitivities and specificities for the detection of PGC on SWMR and conventional MRI were computed with CT as a standard of reference. A McNemar’s paired-sample test was conducted to compare the sensitivity and specificity of conventional MRI and SWMR. As measurements were provided by two independent observers, averages over observers were calculated and analyses were performed average-based. Bland-Altman plots with prediction intervals were produced to display the distribution of measurements and the limits of agreement. As measure outcomes were numeric, the intraclass correlation coefficient (ICC) was used for calculation of intra- and interobserver reliability. According to the commonly cited cut-offs by Cicchetti et al. [31], intra- or interobserver reliability was considered poor for ICC values less than 0.40, fair for values from 0.40 to 0.59, good for values between 0.60 and 0.74 and excellent for values above this threshold. Linear regression was performed to evaluate the correlation between diameter measurements on SWMR, MRI and CT. To determine the confidence intervals for R^2^ a bootstrapping was conducted. A p-value of less than 0.05 was considered statistically significant.

## Results

### Detection rate, sensitivities and specificities for the detection of pineal gland calcifications

All investigated patients received both MRI (including SWMR sequences) and CT imaging of the brain with a time interval of less than three months between the examinations. On CT scans, which were used as the standard of reference for the detection of calcifications, 214 patients showed PGC.

**Fig 1:**
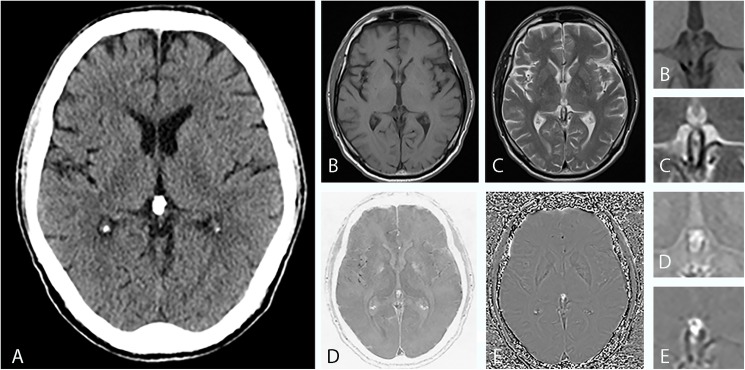
Imaging findings of a 63-year-old man with a calcified pineal gland. (A), CT shows a sharply defined oval-shaped pineal calcification with a diameter of 7 mm. In axial T1-weighted MRI (B) and in axial T2-weighted MRI (C) it is hardly possible to demarcate the calcified area against the surrounding tissue. In conventional MRI, it is not possible to reliably identify the hypointense foci as calcifications. The inverted SWMR magnitude image (D) and the phase image (E) show well-defined focal hyperintensities in the pineal region area. While the image information solely derived from the magnitude image is not superior to conventional MRI sequences, the combination of SWMR magnitude and phase image allows for a clear and reliable identification of diamagnetic calcifications. Magnified images are provided for (B), (C), (D) and (E). As CT and MRI of the brain have diverged reference lines with the bicommissural line used as a convenience standard for MRI and the orbitomeatal line used for CT, the slice angles vary slightly.

**Fig 2:**
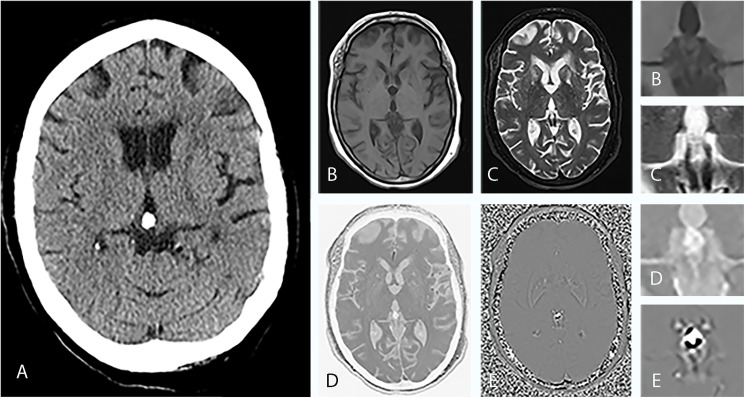
Imaging findings of a 55-year-old woman with a calcified pineal gland. (A), CT shows a sharply defined oval-shaped pineal calcification with a diameter of 11 mm. In axial T1-weighted MRI (B) and in axial T2-weighted MRI (C) it is hardly possible to demarcate the calcified area against the surrounding tissue. In conventional MRI, it is not possible to reliably identify the hypointense foci as calcifications. The inverted SWMR magnitude image (D) and the phase image (E) show well-defined focal hyperintensities in the pineal region area. While the image information solely derived from the magnitude image is not superior to conventional MRI sequences, the combination of SWMR magnitude and phase image allows for a clear and reliable identification of diamagnetic calcifications. Magnified images are provided for (B), (C), (D) and (E). As CT and MRI of the brain have diverged reference lines with the bicommissural line used as a convenience standard for MRI and the orbitomeatal line used for CT, the slice angles vary accordingly.

By combining the SWMR magnitude and the phase image it was possible to identify most of the calcifications (n = 203, 94.9%). Figs 1 and 2 show corresponding examples of PGC and illustrate the clinical applicability of SWMR for the detection of pineal gland-related calcifications compared to conventional MRI sequences. In the 11 cases where SWMR failed to detect calcifications this was due to small lesion size (1.5 ± 1.1 mm) in combination with motion artifacts, which caused a poor image quality leading to a false-negative result. Through assessment of conventional MRI sequences a significantly smaller amount (p = 0.012) of PGC could be differentiated from the surrounding tissue (n = 92, 43,0%), as calcifications appear as hypointense spots both on T_1_ and T_2_ weighted images. Calcifications that were not detected on conventional MRI had a diameter range from 1.2 to 10.9 mm (mean 4.5 ± 2.3 mm). By use of conventional MR imaging, 5 lesions were falsely classified as calcifications because of tissue artifacts.

To evaluate the validity of the diagnostic tests performed, sensitivity and specificity were calculated with respective 95% confidence intervals. Table 1 provides corresponding two-by-two confusion matrixes for SWMR and conventional MRI. While SWMR reached a sensitivity of 95% (95% CI: 91% - 97%) and a specificity of 96% (95% CI: 91% - 99), conventional MRI yielded a sensitivity of only 43.0% (95% CI: 35.0%– 84.1%) and a specificity of 96% (95% CI: 91%– 99%). McNemar’s test confirmed that SWMR showed a sensitivity that was significantly higher compared to conventional MRI sequences (p<0.001). The difference in specificity between SWMR and conventional was not significant (p = 1).

**Table 1:** Two-by-two confusion matrixes for SWMR (A) and conventional MRI (B).

A	CT (reference standard)				B	CT (reference standard)			
**SWMR**		+	-	total	**Conventional MRI**		+	-	total
	+	203	5	208		+	92	5	97
	-	11	127	138		-	122	127	249
	total	214	132	346		total	214	132	346

### Intra- and interobserver correlation

**Fig 3:**
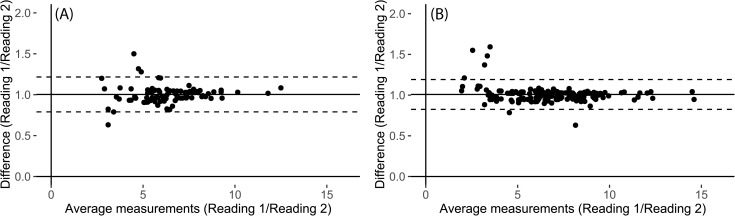
**Bland–Altman plots for the assessment of intraobserver variability for diameter measurements of calcifications in meningiomas in conventional MRI (A) and SWMR (B).** The mean ratio was 1.00 for conventional MRI (CI: 0.79 to 1.22) and 1.01 for SWMR magnitude images (CI: 0.82 to 1.19). The mean ratio of the data is illustrated by the central horizontal line. Upper and lower reference lines show the upper and lower limits of agreement (95% confidence intervals).

**Fig 4:**
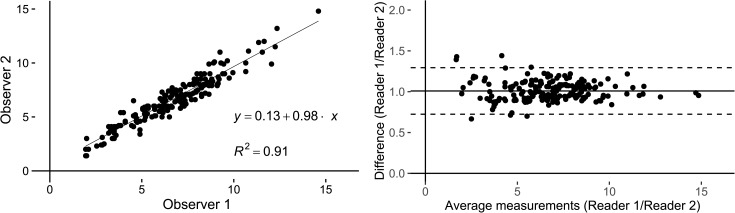
Linear regression and Bland–Altman plot for the assessment of interobserver variability for diameter measurements of calcifications in meningiomas in SWMR. Diameter measurements show an excellent correlation (R^2^ = 0.91) with a mean ratio of 1.00 (CI: 0.72 to 1.29) between calcification measurements of the two readers in SWMR magnitude images. The mean ratio of diameter measurements of readers 1 and 2 is illustrated by the central horizontal line. Upper and lower reference lines show the upper and lower limits of agreement (95% confidence intervals).

Intraobserver agreement on calcification diameters in both CT and SWMR was excellent (ICC = 0.989, p <0.0001 and ICC = 0.984, p <0.0001). Intraobserver agreement for conventional MRI was also excellent (ICC = 0.960, p <0.0001). The visual illustration of intraobserver agreement is provided by the Bland-Altman-plots in Fig 3. Interobserver agreement for diameter measurements in SWMR was also excellent (ICC = 0.954, p <0.0001). On conventional MRI, there was a good intraclass agreement (ICC = 0.712, p<0.0001). Linear regression analysis confirmed a strong correlation between SWMR measurements of two readers (R^2^ = 0.910, p<0.0001, 95% CI: 0.884, 0.932) and a slightly poorer but still good correlation between the two reader measurements in conventional MRI (R^2^ = 0.808, p = 0>0.0001, 95% CI: 0.714, 0.876). Fig 4 provides a linear regression and Bland–Altman plot for the assessment of interobserver variability in SWMR. Compared to standard T_1_ and T_2_ weighted sequences, SWMR showed a considerably higher intra- and interobserver correlation.

### Analysis of calcification diameter

**Fig 5:**
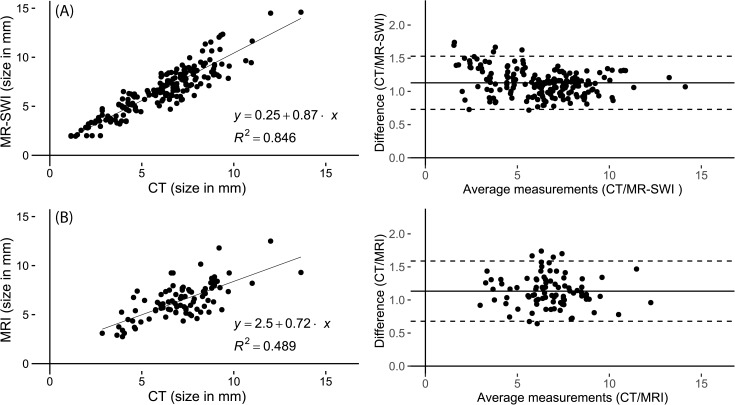
**Linear regression and Bland-Altman plot of the difference between diameter measurements of calcifications in CT and SWMR (A) and of the difference between diameter measurements of calcifications in CT and MRI T1 and T2 weighted images (B).** Diameter measurements show a strong correlation (R^2^ = 0.85) between SWMR magnitude images and the reference standard CT with a mean difference of 1.13 (CI: 0.73 to 1.53). In comparison, correlation between MRI T1 and T2 weighted images and CT is only moderate (R^2^ = 0.49) with a mean difference of 0.92 (CI: 0.53 to 1.30). The mean difference of the data is illustrated by the central horizontal line. Upper and lower reference lines show the upper and lower limits of agreement (95% confidence intervals).

The level of agreement between the imaging modalities SWMR, conventional MRI and CT-based size measurements was illustrated with Bland-Altman plots and the strength of agreement was interpreted based on the intraclass coefficient. Diameter correlations revealed an excellent agreement between SWMR and CT (ICC = 0.881, p <0.0001) and a good to excellent agreement between conventional MRI sequences and CT (ICC = 0.745, p <0.0001). Fig 5 provides the corresponding Bland-Altman plots and linear regression graphs. Correlation was measured by use of a linear regression analysis for the association between two variables, whereby a strong correlation between SWMR and CT was confirmed (R^2^ = 0.844, p <0.001). Correlation between conventional MRI sequences and CT was moderate (R^2^ = 0.48, p <0.001). SWMR showed a slight overestimation of calcification diameters, which, however, did not reach significance level (SWMR: (SWMR: 6.5 ± 2.5 mm; CT: 5.9 ± 2.4 mm, p = 0.02).

## Discussion

The present study shows that using a combination of inverted magnitude and phase images enables the reliable detection of calcifications of the pineal gland without making use of ionizing radiation. The majority of the calcifications identified on CT as the standard of reference could be recognized as calcifications by combining magnitude and phase images with excellent intra- and interobserver reliability and a slight, but not significant, overestimation of diameters. In contrast, conventional MRI sequences did not allow for a reliable identification of calcifications and showed a poor diagnostic performance. Because pineal calcification might lead to a decrease in melatonin production by calcified glands [32], the radiation-free detection of pineal calcifications by SWMR may help to identify patients with an increased risk for the development of neurodegenerative diseases. Furthermore, the assessment of calcifications in the pineal region might also facilitate narrowing differential diagnosis of pineal region tumors, as some of the tumors show unique patterns of calcification.

### Current assessment of PGC

With the development of computed tomography technology, it has been possible to accurately detect the localization and extent of intracranial calcifications, such as choroid plexus, habenular, basal ganglia or pineal gland calcifications, to which many clinical or pathological entities have been linked, e.g. Alzheimer’s or Parkinson’s disease [2, 14]. More recent data from a single retrospective hospital-based study suggested that PGC might also be associated with stroke [33]. However, this was challenged in a subsequent study [34]. With regard to pineal gland calcifications in pediatric patients, one study also found that pineal calcification in childhood appeared to be associated with the development of pediatric primary brain tumors [35].

The incidence of PGC reported in the literature varies depending on the age of the population, its ethnic and geographical makeup and might also vary depending on the use of computed tomography versus skull radiography, as phantom studies showed that cranial CT is up to 15 times more sensitive than skull radiography in the detection of intracranial calcifications [36]. Generally, calcifications of the pineal gland are one of the most common forms of intracranial calcifications with former studies based on computed tomography reporting a prevalence between 68.5% and 75.1% [15, 16, 37]. In all population groups, calcification of the pineal gland was found to increase with age. The findings of the present study regarding the approximate incidence of PGC and its higher prevalence in older age are consistent with the literature [14, 16].

The pineal gland is considered central to chronopharmacology with multiple additional properties such as acting immune-enhancing and cytoprotective [38]. Both the pineal volume and the circadian rhythm of melatonin excretion show a high interindividual variability. Recent years have witnessed an increasing interest in studies regarding the impact of PGC on decreased secretory activity of the gland and on specific pathological entities such as Alzheimer’s disease [2]. Mahlberg et al. suggested, that PGC was significantly higher in patients with Alzheimer’s disease compared to others types of cognitive impairment and that PGC might contribute to the pathogenesis of Alzheimer’s disease by reflecting a reduced level of crystallization inhibitors [2, 4]. With regard to the association between plasma melatonin and uncalcified solid pineal tissue, Liebrich et al. reported that uncalcified pineal functional volume as derived from MRI was linked to the hormonal function of the pineal gland [32, 39]. On the other hand, there has also been a study suggesting that the levels of melatonin did not significantly differ by presence of cysts or calcification [40]. Consequently, it is not yet fully resolved whether there is an association between the incidence of PGC and reduced melatonin excretion.

### Pineal region tumors

The pineal region also plays host to several masses or tumors, the most common being pineal cysts, germinomas or pineocytomas [10]. For pineal mass detection and assessment, MRI, with its excellent soft-tissue contrast and multiplanar imaging abilities, is the modality of choice. However, conventional MRI sequences do not allow for a reliable assessment of calcifications. While pineal tumors can be difficult to differentiate, each of the lesions may have unique imaging characteristics such as tumor-specific patterns of calcifications [13]. Pineal parenchyma tumors usually show “exploded” calcifications, which are dispersed to the periphery, whereas pineal germinomas tend to engulf the calcification [12, 13]. As a consequence, the addition of SWMR sequences to the MRI standard protocol might prospectively also be able to facilitate the narrowing of differential diagnosis with regard to pineal region masses.

### Use of SWMR to evaluate pineal gland calcifications

Advances in MRI techniques for the evaluation of changes in magnetic susceptibility eventually culminated in the development of SWMR, enabling a reliable detection of calcified lesions in MRI possible [17, 18, 41]. Magnetic susceptibility is a measure of the magnetic properties of a material placed in a magnetic field. Diamagnetic (e.g. calcifications), ferromagnetic (e.g. iron) or paramagnetic (e.g. hemosiderin) substances distort the local magnetic field and cause a phase alteration of the local tissue, which results in a loss of signal. Paramagnetic compounds line up with the external magnetic field, whereas diamagnetic substances align in the opposite direction. The introduction of new filtering techniques has made it possible to differentiate between calcifications, hemorrhage and other causes of susceptibility changes. While calcifications cause a negative phase shift with a hyperintensity on phase-filtered images, hemosiderin or desoxyhemoglobin result in a positive phase shift and a signal loss on phase images, provided that an appropriate echo time is selected [17, 18, 24, 41].

In the present study, it was possible to reliably identify most PGCs based on SWMR, using a combination of SWMR magnitude and phase images. Computed tomography was used as a standard of reference. The reconstruction kernel applied (FC26) is a standard reconstruction kernel used in the clinical setting for the detection of calcifications. In 11 cases SWMR failed to accurately detect pineal calcifications. This was due to a combination of poor image quality resulting from motion artifacts and small size/inhomogeneity of the calcifications. Comparable findings in different disease entities have been shown in a series of previous studies addressing the detection of calcification by use of SWMR [25–27], such as, for example, one study which demonstrated the reliable detection of intratumoral calcifications in oligodendrogliomas [42].

### Development of quantitative susceptibility mapping (QSM)

In recent years, there were further developments in magnetic susceptibility imaging regarding the quantification of signal changes. While typical SWI depends on nonlocal and orientation-dependent phase signals, making it challenging to quantify susceptibility changes in phase images, the novel development of quantitative susceptibility mapping is nonlocal and allows for quantitative measurements of magnetic susceptibility [43]. Compared to magnetic susceptibility imaging, which generates a robust contrast based on phase images, QSM computes the susceptibility of each voxel in form of a scalar quantity [43]. Although there still is an ongoing discussion on which algorithms are best suited to achieve a pixel-by-pixel estimate of susceptibility distribution [44], QSM has been shown to have a high sensitivity for the detection and quantification of iron and to demonstrate superb contrast of brain structures [45]. With regard to the detection of calcification, several studies suggest that QSM may be superior to conventional SWI in the identification of subtle calcifications and in differentiating microcalcifications from hemosiderin, at the same time also allowing for a quantification [19, 46]. As a consequence, there is a multitude of future clinical applications for QSM, such as in the diagnosis and longitudinal evaluation of quantitative changes in neurodegenerative diseases [47], multiple sclerosis [48] or the prognosis of neoplasms such as glioblastomas [46].

### Limitations

This study has several potential limitations. Even though pineal calcifications have been linked to various pathologies such as neurodegenerative diseases, so far, no definite association has been proven. Further studies are needed to investigate the clinical significance of pineal gland calcification. Furthermore, it might be argued that we did not use the typical SWI imaging, where calcifications or other causes of susceptibility changes between tissues are highlighted as signal voids with a negative contrast, but an approach using a combination of inverted magnitude images and filtered phase images. However, while SWI has been proven to be well suited for visualization of small susceptibility changes in the cerebrum, we think this approach might be better suited for visualization of relatively large calcifications surrounded by CSF, such as pineal gland calcifications. Conclusions from our data are not applicable to patients with contraindications to MRI.

### Future studies

SWMR was performed on a 1.5 Tesla scanner in the present study. The comparatively lower field strength has the advantage of a higher magnetic field homogeneity and a consecutive reduction of artifacts. While higher field strengths such as 3 Tesla might cause an increase in the overestimation of calcification sizes, they provide a superior signal-to-noise- and contrast-to-noise- ratio with shorter scan times and can consequently further improve detection rates. As a consequence, comparison of 1.5 versus 3 Tesla scanners for susceptibility-weighted imaging might be addressed in future studies.

Another aspect is, that there have been few studies so far, which directly compared SWI and QSM to each other. It would be very interesting for future research to compare both methods to determine their diagnostic performance for various applications.

### Conclusion

Combining SWMR magnitude and phase images allows for the accurate detection and assessment of pineal gland calcification, using non-enhanced CT as standard of reference. Compared to conventional MRI, SWMR offers a considerably higher diagnostic performance.
